# RIC-7 Promotes Neuropeptide Secretion

**DOI:** 10.1371/journal.pgen.1002464

**Published:** 2012-01-19

**Authors:** Yingsong Hao, Zhitao Hu, Derek Sieburth, Joshua M. Kaplan

**Affiliations:** 1 Department of Molecular Biology, Massachusetts General Hospital, Boston, Massachusetts, United States of America; 2 Department of Neurobiology, Harvard Medical School, Boston, Massachusetts, United States of America; 3 Zilkha Neurogenetic Institute, University of Southern California, Los Angeles, California, United States of America; University of California San Diego, United States of America

## Abstract

Secretion of neurotransmitters and neuropeptides is mediated by exocytosis of distinct secretory organelles, synaptic vesicles (SVs) and dense core vesicles (DCVs) respectively. Relatively little is known about factors that differentially regulate SV and DCV secretion. Here we identify a novel protein RIC-7 that is required for neuropeptide secretion in *Caenorhabditis elegans*. The RIC-7 protein is expressed in all neurons and is localized to presynaptic terminals. Imaging, electrophysiology, and behavioral analysis of *ric-7* mutants indicates that acetylcholine release occurs normally, while neuropeptide release is significantly decreased. These results suggest that RIC-7 promotes DCV–mediated secretion.

## Author Summary

Neuropeptides produce prolonged changes in circuit activity that are associated with changes in behavioral states (e.g. mood or appetite); consequently, there is great interest in identifying molecules that are required for neuropeptide secretion. Here we show that a novel neuronal protein RIC-7 promotes neuropeptide secretion in *C. elegans* but has only subtle effects on neurotransmitter secretion. RIC-7 is conserved in several other nematodes; however, homologous proteins are not found in other sequenced genomes. These results suggest that the machinery responsible for neuropeptide secretion evolved more recently than factors that are required for both neurotransmitter and neuropeptide secretion.

## Introduction

Neurons secrete both neuropeptides and neurotransmitters. Neurotransmitters, such as acetylcholine (ACh), are secreted by exocytosis of small clear synaptic vesicles (SVs) whereas neuropeptide secretion is mediated by exocytosis of dense core vesicles (DCVs) [1], [2]. The mechanisms leading to DCV and SV exocytosis are similar in many respects. SVs and DCVs both undergo physical docking to the plasma membrane, requiring Munc18 and syntaxin for docking in both cases [3]–[7]. To become fusion competent, SVs and DCVs must both undergo a priming reaction, which is mediated by priming factors (e.g. Munc13 and CAPS) [8], [9]. Exocytosis of SVs and DCVs are both mediated by assembling complexes between vesicular and plasma membrane SNARE proteins [10], [11]. Finally, calcium-evoked fusion of SVs and DCVs are mediated by distinct calcium sensors, which are thought to be different synaptotagmin isoforms [12].

Beyond these similarities, DCVs and SVs exhibit many important differences. DCVs can be found all along the cell body, dendrites and axons of neurons whereas SVs cluster specifically at active zones of synapses [13]. SVs undergo repeated cycles of exo- and endocytosis at synapses, whereas neuropeptides are only packaged into nascent DCVs in the Golgi [14]. Consequently, DCVs cannot undergo local recycling in axons or dendrites. DCVs release their contents over long timescales (>50 ms) while SV exocytosis occurs more rapidly (<20 ms) [13], [15]. Exocytosis of SVs can be evoked by single action potentials while DCV release typically occurs after more prolonged or repeated depolarizations. These differences imply that different molecules are involved in SV and DCV secretion.

To date, very few proteins have been found that are specifically involved in the secretion of one or the other class of vesicles. UNC-31/CAPS (Calcium-dependent Activator Protein for Secretion) is proposed to promote priming of DCVs but not SVs [16]–[18]. However, a subsequent study showed compelling evidence for SV priming defects in CAPS1 and CAPS2 double knockout mice [19], implying that CAPS is also required for SV priming. Similarly, some studies propose that Munc13 primes SVs but not DCVs [18], while others find Munc13 mutants have exocytosis defects for both SVs and DCVs [20], [21]. *C. elegans* mutants lacking PKC-1, a PKCε ortholog, had significant defects in DCV release but little effect on SV release [20]. Identifying new genes that differentially regulate SV or DCV release will provide new insights into the mechanisms underlying these two forms of secretion.

In *C. elegans*, the acetylcholinesterase inhibitor aldicarb has been widely used to study neuromuscular signaling in live animals. Aldicarb treatment causes acetylcholine (ACh) to accumulate in the synaptic cleft at NMJs, resulting in an acute paralysis of treated animals. Mutations or RNAi treatments that reduce ACh release confer resistance to aldicarb-induced paralysis, whereas those that stimulate ACh secretion enhance aldicarb sensitivity [22], . We previously showed that neuropeptides also regulate aldicarb responsiveness [24], [25]. Inactivation of genes encoding proneuropeptide processing enzymes [for example, *egl-3* prohormone convertase (PC2), *egl-21* carboxypeptidase E (CPE), *sbt-1* 7B2, and *nep-1* neprilysin], proneuropeptides (*ins-22, ins-31, flp-1, nlp-12*), neuropeptide receptors (*fshr-1*) all cause aldicarb resistance [24], [25]. These results suggested that new genes that are required for DCV secretion could be identified through screens for aldicarb resistant mutants.

In a screen for mutations that suppress the aldicarb hypersensitivity of *dgk-1* diacylglycerol kinase (DAGK) mutants, we isolated a new allele of the *ric-7* gene. Here we show that *ric-7* encodes a novel nematode specific protein that is required for neuropeptide secretion.

## Results

### RIC-7 functions in cholinergic neurons for aldicarb responsiveness

**Figure 1:**
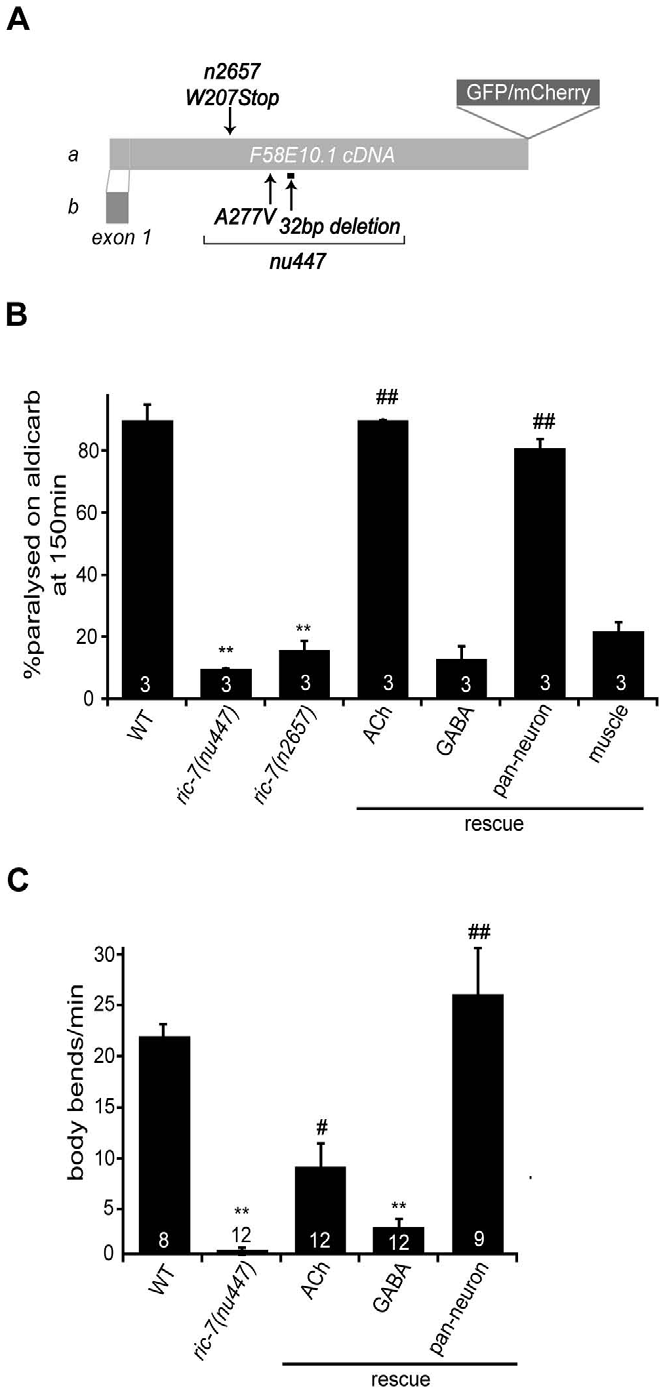
Aldicarb response and locomotion defects in *ric-7* mutants. (A) A schematic illustrating the *ric-7* gene structure is shown. Deleted regions (bar), point mutations (arrows), and the site utilized for GFP/mCherry tagging are indicated. (B–C) Aldicarb-induced paralysis (B) and locomotion rate (C) are compared for the indicated genotypes. The number of trials (∼20 animals/trial, B) or the number of animals analyzed (C) are indicated for each genotype. For rescue experiments, *ric-7* transgenes are indicated as follows: ACh (*unc-17* promoter), GABA (*unc-25* promoter), pan-neuron (*snb-1* promoter), muscle (*myo-3* promoter). Values that differ significantly from wild type (**, p<0.001, Student's t-test) and from *ric-7* mutants (#, p<0.01; ##, p<0.001, Student's t-test) are indicated. Error bars indicate SEM.

To identify new genes required for neuromuscular function, we screened for mutations that suppress the aldicarb hypersensitivity defect of *dgk-1* DAGK mutants. One suppressor (*nu447*) significantly decreased the aldicarb hypersensitivity of *dgk-1* mutants. The *nu447* mutation mapped close to the *ric-7* gene, which was identified in prior screens for aldicarb resistant mutants [22]. We found that *nu447* and *ric-7(n2657)* both correspond to mutations in F58E10.1 gene (Figure 1A), hereafter referred to as the *ric-7* gene. The *ric-7* locus encodes two isoforms (A and B) that differ only in their first exon. Orthologs of *ric-7* are observed in several other nematodes, but homologous genes are not detected in other metazoans. The predicted RIC-7 protein does not contain any previously described structural domains.

**Figure 2:**
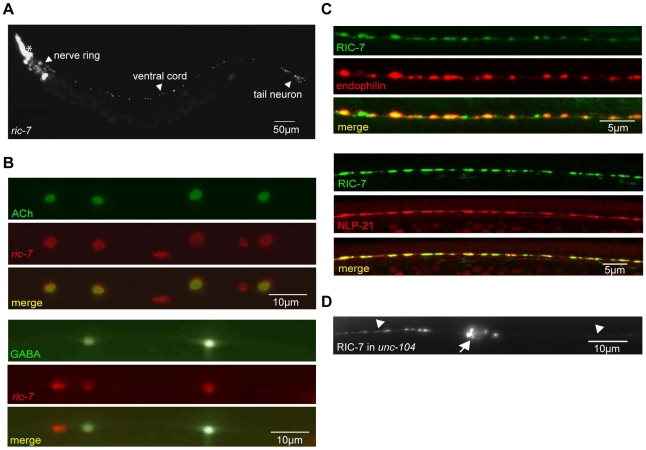
Expression pattern of RIC-7. (A) The *ric-7* promoter expresses nuclear localized Cherry (HIS-24::wCherry) primarily in the nervous system of an adult worm. Anterior is left; ventral is up. The asterisk indicates fluorescence encoded by a co-injection marker. (B) The *ric-7* promoter is expressed in cholinergic (top panel) and GABAergic (bottom panel) motor neurons in the ventral cord. Cell bodies of cholinergic (*unc-17* promoter) and GABAergic (*unc-30* promoter) neurons were identified by expression of the indicated GFP reporter constructs. (C) Distribution of RIC-7, a synaptic vesicle marker (UNC-57 Endophilin) (top panel), and a DCV marker (NLP-21) (bottom panel) are compared in the dorsal cord axons of cholinergic motor neurons. (D) Distribution of RIC-7::GFP in cholinergic motor neurons of *unc-104* KIF1A mutants. Cell bodies (arrow) and ventral cord processes (arrow heads) are indicated.

Animals homozygous for *nu447* or *n2657* were resistant to the paralytic effects of aldicarb (Figure 1B). To determine whether RIC-7 functions in motor neurons for aldicarb responsiveness, we constructed a *ric-7* transcriptional reporter. The resulting construct expressed GFP in many neurons, including both cholinergic and GABAergic motor neurons (Figure 2A–2B). Transgenes expressing either RIC-7A or B isoforms in all neurons (with the *snb-1* promoter), and those expressing RIC-7B in cholinergic neurons (with the *unc-17* promoter) rescued the aldicarb sensitivity defect of *ric-7* mutants (Figure 1B). By contrast, expressing RIC-7B in GABAergic neurons (*unc-25* promoter) or in muscles (*myo-3* promoter) did not rescue the aldicarb phenotype of *ric-7* mutants (Figure 1B). These results suggest that RIC-7 activity is required in cholinergic neurons for aldicarb responsiveness.

Animals lacking *ric-7* also had decreased locomotion rates (Figure 1C). This locomotion defect was fully rescued by *ric-7* transgenes expressed in all neurons (using the *snb-1* promoter) whereas partial rescue was observed with transgenes expressed in cholinergic or GABAergic neurons (Figure 1C). The morphology of motor neuron axons and NMJs appeared superficially normal in *ric-7* mutants (Figure 4C and data not shown), suggesting that these motor defects were unlikely to be caused by changes in neural development.

### RIC-7 is targeted to presynaptic terminals

We expressed GFP-tagged RIC-7 constructs in the cholinergic DA neurons (using the *unc-129* promoter). The RIC-7::GFP protein was localized in a punctate distribution in both cell bodies and dorsal cord axons. The majority of RIC-7 puncta co-localized with an SV marker (mCherry-tagged Endophilin) [26], suggesting that RIC-7 is targeted to synapses (Figure 2C). We also compared the distribution of GFP-tagged RIC-7 with a mCherry-tagged neuropeptide, NLP-21. Whereas RIC-7 showed partial overlap with endophilin, nearly complete co-localization was observed with NLP-21 (Figure 2C). Several results suggest that the co-localization of RIC-7 with DCVs was not mediated by physical association of RIC-7 with nascent DCVs. First, RIC-7 synaptic localization was not disrupted in *unc-104* mutants (Figure 2D), which lack the KIF1A motor responsible for anterograde transport of SVs and DCVs. Thus, RIC-7 is not co-transported to synapses with immature DCVs or SVs. Second, RIC-7 lacks a predicted signal peptide sequence and GFP-tagged RIC-7 did not produce fluorescence in coelomocytes, both of which indicate that RIC-7 is not translocated into SVs or DCVs (data not shown). These results support the idea that RIC-7 functions in the cytoplasm at presynapses and could play a relatively direct role in regulating SV or DCV secretion.

### Muscle responsiveness to ACh and GABA is unaltered in *ric-7* mutants

**Figure 3:**
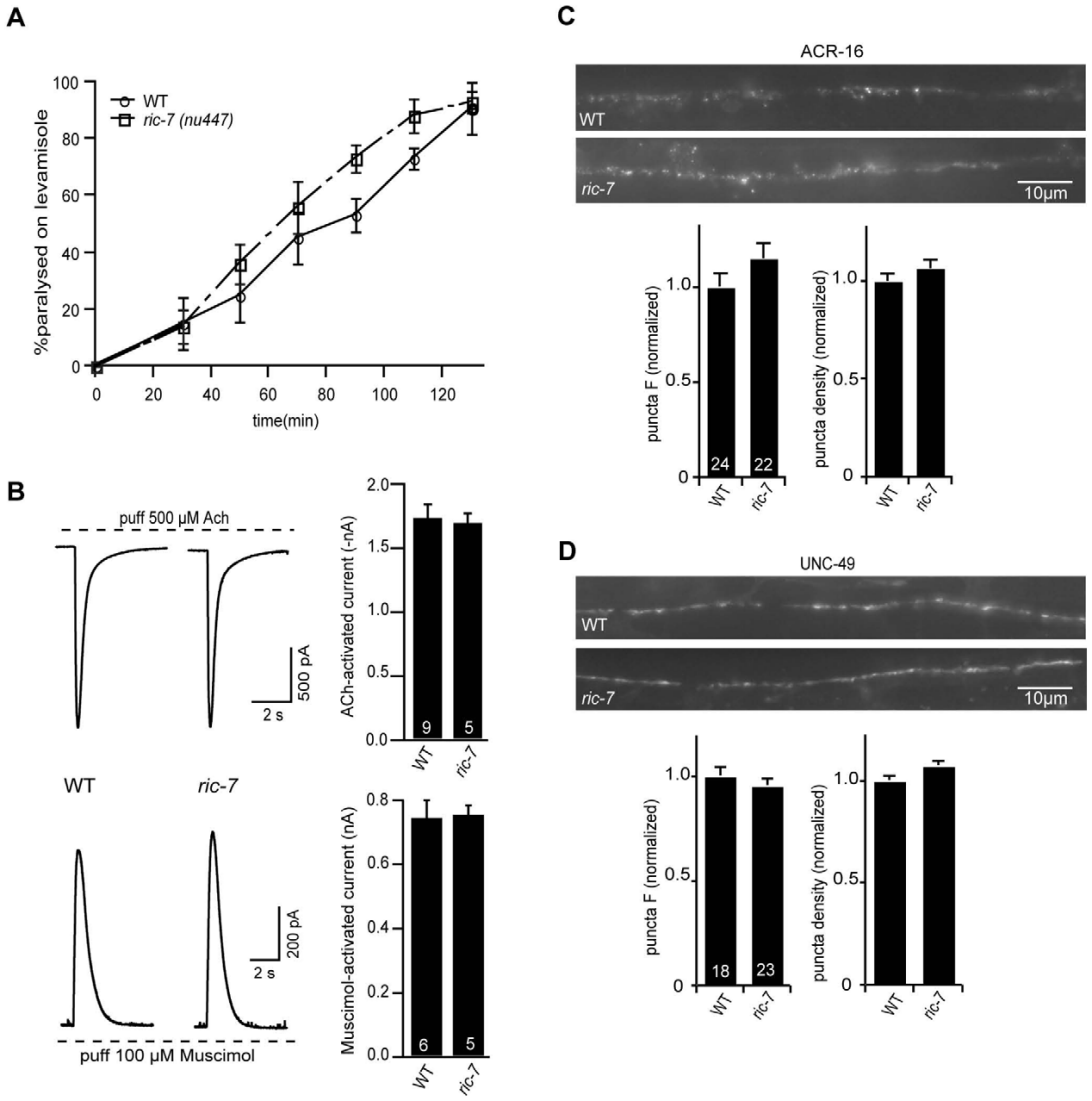
Body muscle responses to ACh and GABA are unaltered in *ric-7* mutants. (A) Time course of levamisole (200 µM) induced paralysis is shown for wild type and *ric-7(nu447)* adults. Three trials (∼20 animals/trial) were performed for each genotype. (B) ACh (top) and Muscimol (bottom)-activated currents were recorded from body wall muscles of *ric-7* and wild type adults. Representative responses (left) and summary data (right) are shown. Wild type and *ric-7* mutant responses were not significantly different. (C–D) Representative images (above) and summary data (below) are shown for ACR-16::GFP (C) and UNC-49::GFP (D) expressed in body muscles of wild type and *ric-7* adults (using the *myo-3* promoter). No significant differences were observed. The number of animals analyzed is indicated for each genotype (panels B–D). Error bars indicate SEM.

We did several experiments to test the effects of RIC-7 on the responsiveness of body muscles to neuromuscular agonists (Figure 3). First, the sensitivity of *ric-7* mutants to the paralytic effects of the nicotinic agonist levamisole was similar to that of wild type controls (Figure 3A). Second, we recorded body wall muscle currents evoked by application of ACh or the GABA agonist muscimol. In both cases, the amplitude of agonist-evoked current in *ric-7* mutant body muscles was not significantly different from that observed in wild type controls (Figure 3B). Third, the fluorescent intensities of GFP-tagged ACR-16 ACh receptor (Figure 3C) and UNC-49 GABA_A_ receptor (Figure 3D) puncta in the nerve cord were unaltered in *ric-7* mutants, indicating that the abundance of post-synaptic receptors at NMJs was normal. Thus, the effect of RIC-7 on aldicarb responsiveness is unlikely to be caused by altered agonist responsiveness of body muscles.

### ACh release occurs normally in *ric-7* mutants

**Figure 4:**
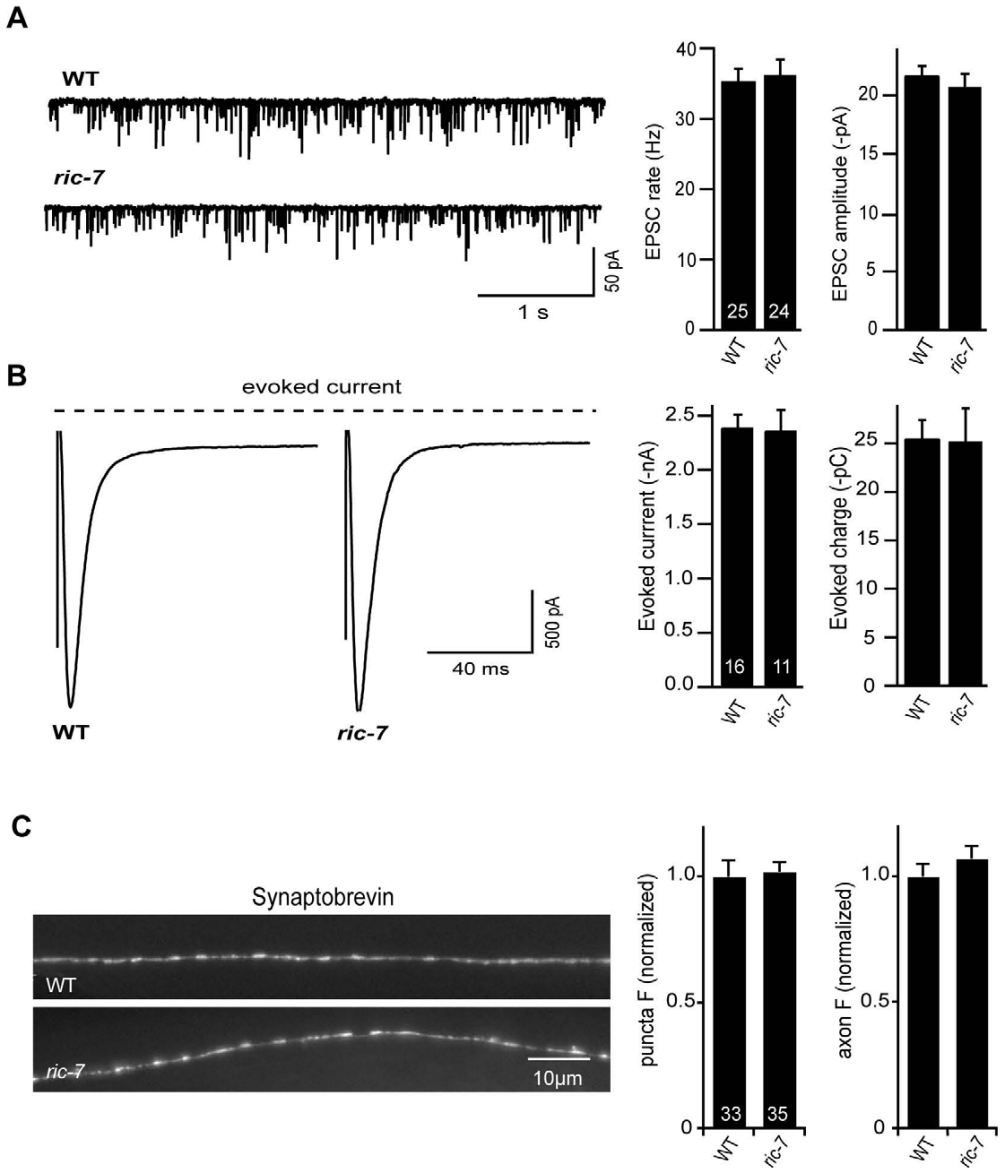
Baseline ACh release is unaltered in *ric-7* mutants. Endogenous EPSCs (A) and stimulus-evoked EPSCs (B) were recorded from body wall muscles of wild type and *ric-7(nu447)* adults. Representative traces of endogenous EPSCs (A), averaged traces of stimulus-evoked responses (B), and summary data for both are shown. The number of animals analyzed is indicated for each genotype. No significant differences were observed. (C) Representative images (left) and summary data (right) are shown for GFP-tagged SNB-1 in dorsal cord axons of cholinergic motor neurons (expressed with the *unc-129* promoter) in wild type and *ric-7* adults. The number of animals analyzed is indicated for each genotype. No significant differences were observed. Error bars indicate SEM.

The aldicarb resistance phenotype observed in *ric-7* mutants could be caused by decreased ACh secretion, increased GABA secretion, or decreased neuropeptide secretion, as aldicarb resistance would be expected in all three scenarios [25], [27], [28]. To assay ACh secretion more directly, we recorded excitatory post-synaptic currents (EPSCs) from body muscles (Figure 4). We found that the rate and amplitude of endogenous EPSCs, i.e. SV fusions evoked by the endogenous activity of motor neurons, in *ric-7* mutants were similar to those found in wild type animals (Figure 4A). In addition, the amplitude of stimulus-evoked EPSCs in *ric-7* mutants was not significantly different from wild type (Figure 4B). Therefore, baseline ACh secretion occurs normally at cholinergic NMJs in *ric-7* mutants.

To further analyze the cholinergic NMJs, we examined the distribution of GFP-tagged Synaptobrevin (GFP::SNB-1) in motor neurons. Changes in the distribution of GFP::SNB-1 are correlated with changes in SV exo- and endocytosis. SNB-1 puncta intensity is correlated with the number of SVs at presynaptic elements [24], [29] whereas puncta density is a measure of synapse density. Neither the fluorescent intensity nor the density of SNB-1 puncta in the cholinergic axons of *ric-7* mutants were significantly different from that observed in wild type controls (Figure 4C). Taken together, these results suggest that defects in baseline ACh secretion are unlikely to account for the aldicarb resistance of *ric-7* mutants.

### RIC-7 promotes neuropeptide secretion

**Figure 5:**
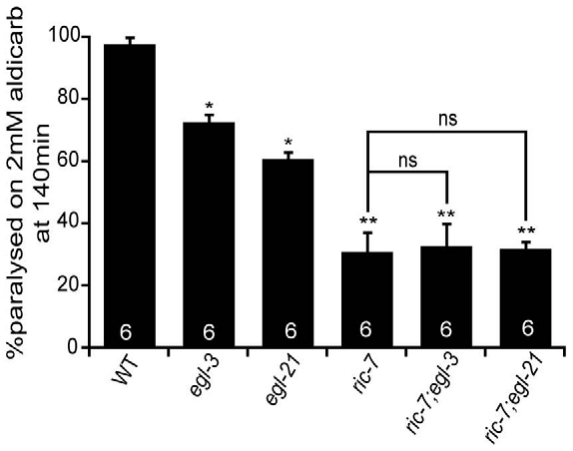
Neuropeptide processing mutations and The paralytic response to aldicarb treatment was analyzed in strains containing mutations that inactivate pro-neuropeptide processing enzymes (*egl-3* PC2 and *egl-21* CPE), or those inactivating RIC-7. The number of trials (∼20 animals/trial) is shown for each genotype. Values that differ significantly from wild type (*, p<0.01; **, p<0.001, Students t-test) are indicated. Error bars indicate SEM. Values that are not significantly different are indicated (ns).

Aldicarb resistance could also be caused by defects in neuropeptide signaling [25]; therefore, we next examined *ric-7* mutants for changes in neuropeptide signaling. First, we analyzed the aldicarb responsiveness of *ric-7* double mutants containing mutations in neuropeptide signaling components. If changes in neuropeptide action contribute to RIC-7's effects on aldicarb responsiveness, then we would expect that *ric-7* mutations would occlude the effect of neuropeptide signaling mutations on aldicarb resistance. Consistent with this idea, *ric-7* double mutants carrying mutations in either of two proneuropeptide processing enzymes (*egl-21* CPE and *egl-3* PC2) had aldicarb resistance that was similar to that observed in *ric-7* single mutants (Figure 5). These results support the idea that RIC-7 regulation of neuropeptide secretion contributes to the effects of *ric-7* mutations on aldicarb responsiveness.

**Figure 6:**
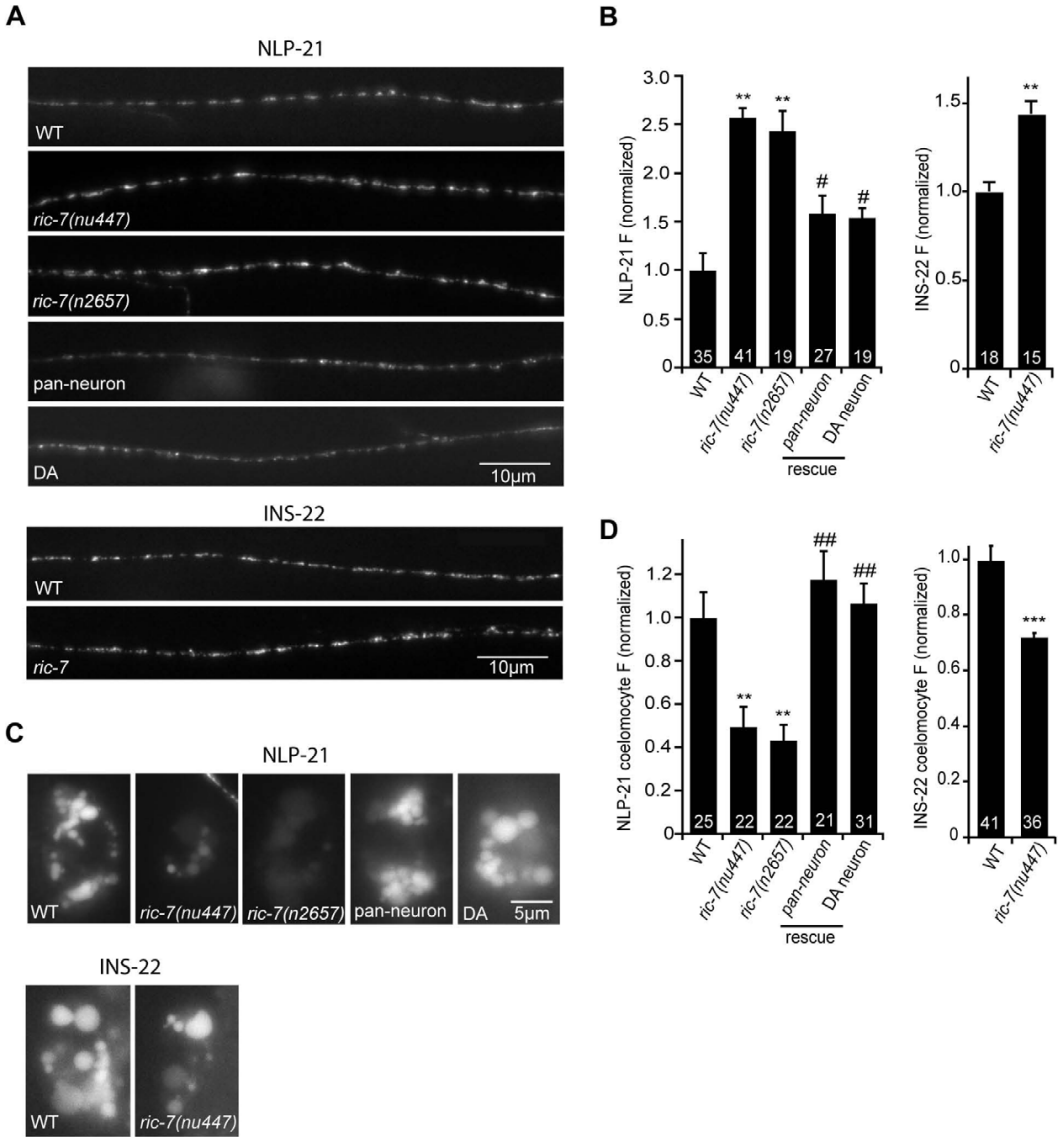
RIC-7 promotes neuropeptide release. YFP-tagged NLP-21 and INS-22 were expressed in cholinergic motor neurons using the *unc-129* promoter. Representative images (A) and summary data (B) are shown for NLP-21 (top) and INS-22 (bottom) fluorescence in dorsal cord axons of the indicated genotypes. The number of animals analyzed is indicated for each genotype. (C–D) Representative images (C) and summary data (D) are shown for NLP-21 and INS-22 fluorescence in coelomocytes of the indicated genotypes. The number of animals analyzed is indicated for each genotype. Values that differ significantly from wild type (**, p<0.001, ***, p<0.0001 Students t-test) and from *ric-7* mutants (#, p<0.01, ##, p<0.001, Students t-test) are indicated. Error bars indicate SEM. For rescue experiments, *ric-7* transgenes are as follows: pan-neuron (*snb-1* promoter), DA neuron (*unc-129* promoter).

To further address the role of RIC-7 in neuropeptide secretion, we analyzed the secretion of YFP-tagged neuropeptides (Figure 6). For this analysis, we selected two proneuropeptides, NLP-21 and INS-22, which encode FMRFamide related peptides (FaRPs) and an insulin-like growth factor, respectively. When NLP-21::YFP or INS-22::YFP are expressed in the cholinergic DA motor neurons (using the *unc-129* promoter), puntate fluorescence is detected in dorsal cord axons and in coelomocytes (Figure 6A and 6C). We previously showed that the axonal puncta fluorescence corresponds to secretory granules containing these proneuropeptides while the coelomocyte fluorescence corresponds to secreted neuropeptides that have been endocytosed [20], [30]. In *ric-7* mutants, NLP-21 coelomocyte fluorescence was significantly decreased (∼50%, p<0.001) (Figure 6C–6D) whereas the NLP-21 puncta fluorescence intensity in dorsal cord axons was significantly increased (>2-fold, p<0.001) (Figure 6A–6B). The coelomocyte and axonal NLP-21 fluorescence defects were both rescued by *ric-7* transgenes expressed in the DA neurons. Similar changes in axonal puncta fluorescence and coelomocyte fluorescence was observed for a second proneuropeptide (INS-22) in *ric-7* mutants (Figure 6). These results suggest that *ric-7* mutants have decreased neuropeptide secretion, which results in an accumulation of DCVs in motor axons.

### RIC-7 is required for NLP-12–mediated enhancement of ACh release

**Figure 7:**
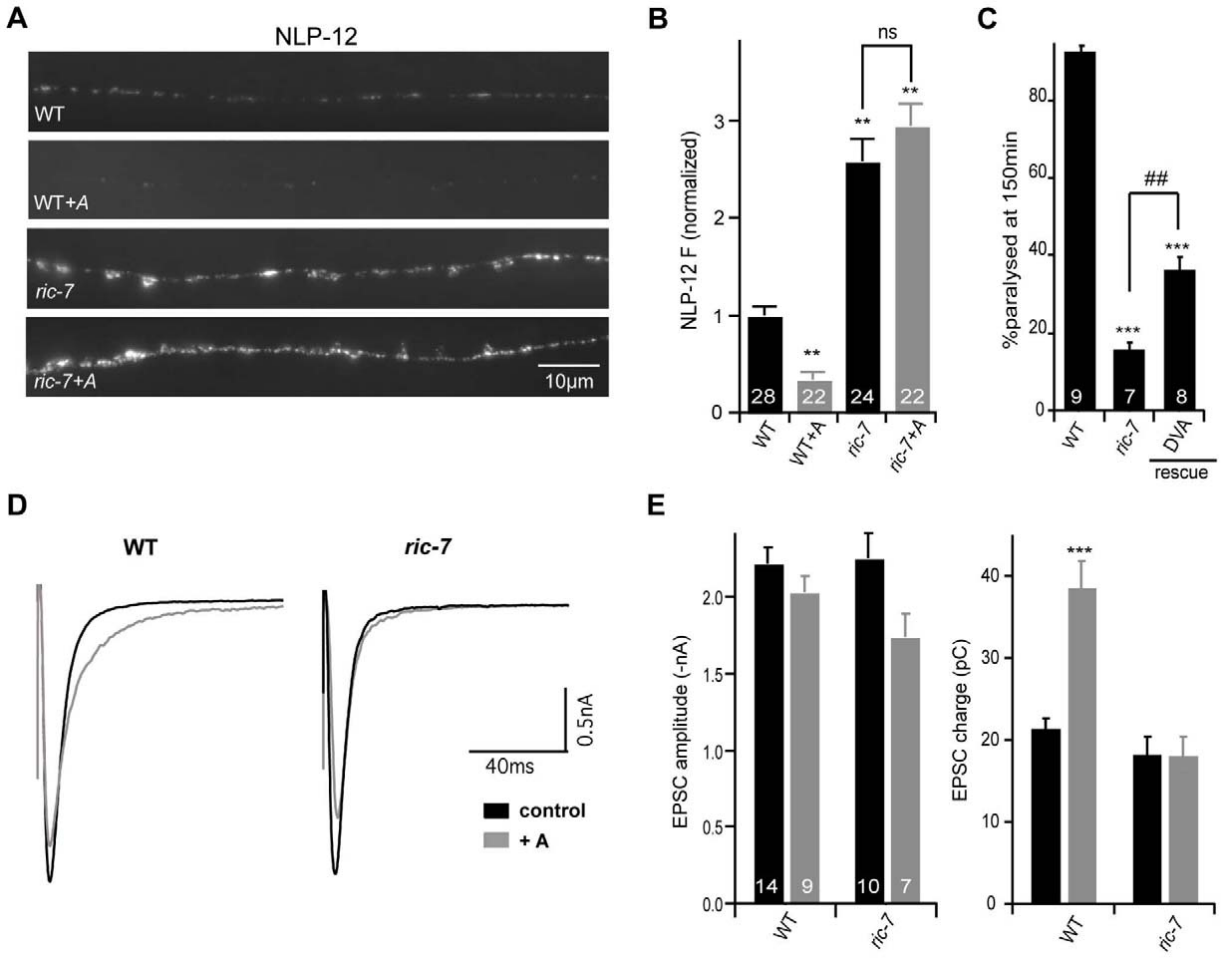
NLP-12 secretion is decreased in *ric-7* mutants. (A) YFP-tagged NLP-12 was expressed in DVA neurons (using the *nlp-12* promoter). Representative images (A) and summary data (B) are shown for NLP-12 puncta fluorescence in the indicated genotypes. The number of animals analyzed is indicated for each genotype. (C) Aldicarb-induced paralysis was quantified for the indicated genotypes. The number of trials (∼20 animals/trial) is indicated for each genotype. (D–E) Stimulus evoked EPSCs were recorded from adult body wall muscles of the indicated genotypes. Recordings were done after a 60 minute aldicarb pre-treatment (gray) or in untreated controls (black). Averaged evoked responses (D) and summary data (E) are shown. The number of animals analyzed is indicated for each genotype. Values that differ significantly from wild type (**, p<0.001, ***, p<0.0001, Students t-test) and from *ric-7* mutants (##, p<0.001, ###, p<0.0001 Students t-test) are indicated. Error bars indicate SEM. For rescue experiments, *ric-7* transgenes are as follows: DVA (*nlp-12* promoter).

Inactivation of NLP-21 and INS-22 by RNAi does not produce strong locomotion or aldicarb resistance defects [24]; consequently, NLP-21 and INS-22 are unlikely to be the only neuropeptides involved in the Ric-7 locomotion and aldicarb-resistance phenotypes. We recently identified NLP-12 as a neuropeptide that plays a critical role in regulating both locomotion rate and aldicarb-induced paralysis [31]. NLP-12 is expressed in a proprioceptive neuron (DVA) that is activated by body muscle contractions [32], [33]. If Ric-7 aldicarb resistance and locomotion defects are caused by decreased neuropeptide release, we would expect that NLP-12 secretion from DVA would be diminished in *ric-7* mutants. We did several experiments to test this idea. First, we analyzed the effect of *ric-7* mutations on secretion of YFP-tagged NLP-12 from DVA neurons (Figure 7A–7B). In wild type animals, aldicarb treatment significantly decreased NLP-12 puncta fluorescence in DVA axons (indicating increased NLP-12 secretion), which is most likely caused by activation of DVA stretch receptors by muscle contraction [31]. By contrast, in *ric-7* mutants, aldicarb had no effect on NLP-12 puncta fluorescence, indicating that RIC-7 is required for aldicarb-evoked NLP-12 secretion.

Second, if the NLP-12 secretion defect contributes to the Ric-7 aldicarb resistance phenotype, we would expect that RIC-7 expression in DVA neurons would be sufficient to alter aldicarb responsiveness. Consistent with this idea, the aldicarb responsiveness of *ric-7* mutants was significantly improved by transgenes expressing RIC-7 in DVA neurons (Figure 7C). This result is consistent with the preceding rescue data (Figure 1B) because the *unc-17* promoter (which also rescued the Ric-7 aldicarb defect) is expressed in DVA neurons (data not shown). Collectively, these data suggest that proper aldicarb responsiveness requires RIC-7 function in multiple neuron classes because RIC-7 expression in a single cholinergic neuron (DVA) produced partial rescue of the aldicarb defect whereas complete rescue was obtained following expression in all cholinergic neurons (with the *unc-17* promoter) (Figure 1B).

Third, we analyzed evoked ACh release following aldicarb treatment. In wild type animals, a 60 minute pre-treatment with aldicarb significantly increases the total synaptic charge occurring during an evoked response (Figure 7D–7E) [31]. This effect is eliminated in *egl-3* PC2 mutants and in *nlp-12* mutants [31]. Thus, aldicarb enhancement of evoked ACh release can be utilized to assess changes in endogenous NLP-12 secretion. Aldicarb's effect on evoked ACh release was also eliminated in *ric-7* mutants (Figure 7D–7E). Collectively, these results strongly support the idea that RIC-7 acts in DVA neurons to promote secretion of endogenous NLP-12, and that this contributes to the Ric-7 aldicarb-resistance defect.

### GABA transmission is altered in *ric-7* mutants

**Figure 8:**
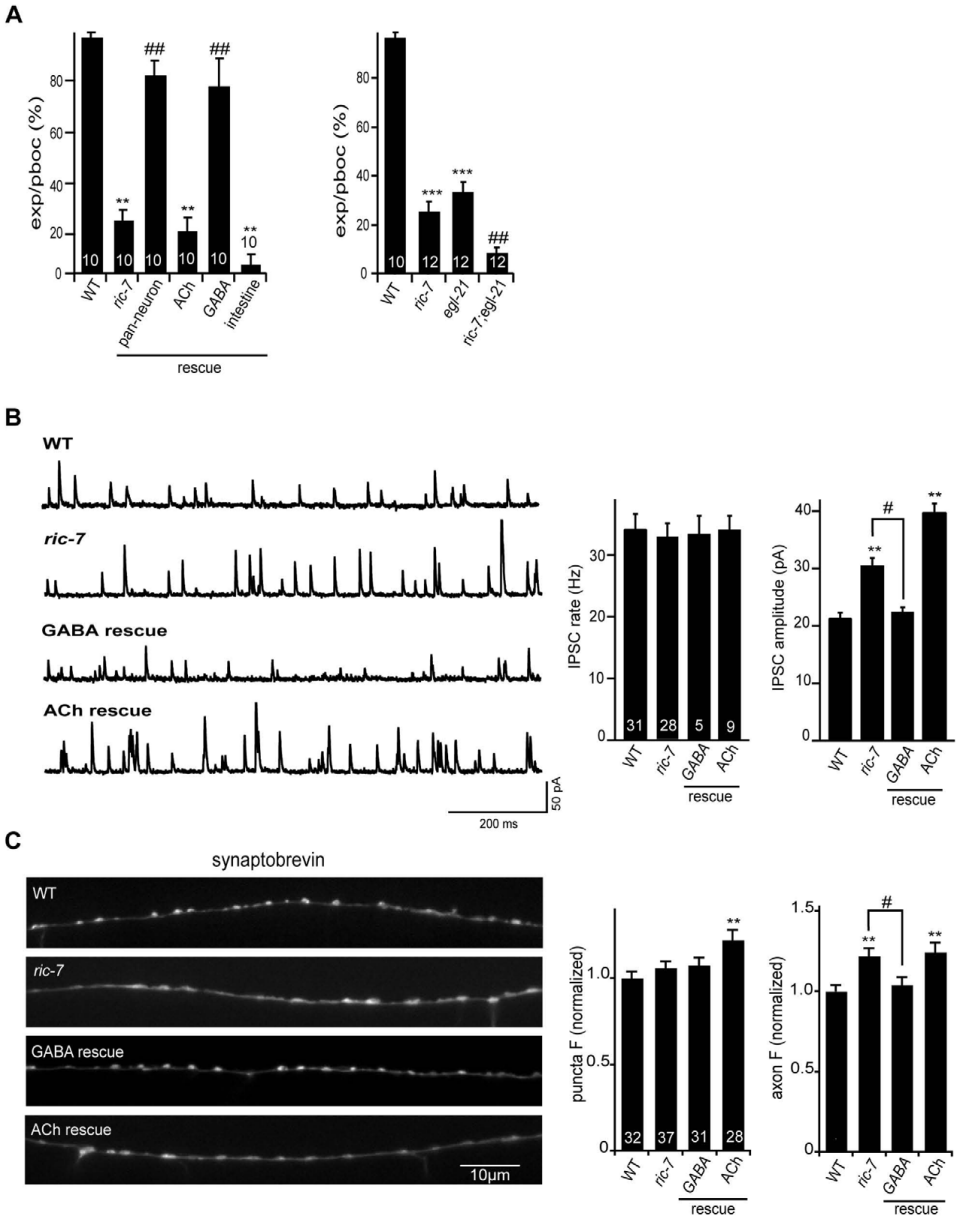
Analysis of GABA transmission in *ric-7* mutants. (A) Intestinal muscle contractions during the defecation motor program (quantified as expulsions/pBoc) were analyzed in the indicated genotypes. (B) Endogenous IPSCs were recorded from adult body wall muscles of the indicated genotypes. Representative traces (left), and summary data (right) are shown. The number of animals analyzed is indicated for each genotype. (C) Representative images (left) and summary data (right) for GFP::SNB-1 (expressed by the *unc-25* promoter) in dorsal cord axons of the indicated genotypes. The number of animals analyzed is indicated for each genotype. Values that differ significantly from wild type (**, p<0.001, ***, p<0.0001 Students t-test) and from *ric-7* mutants (#, p<0.05, ##, p<0.001, ###, p<0.0001 Students t-test) are indicated. Error bars indicate SEM. For rescue experiments, *ric-7* transgenes are as follows: ACh (*unc-17* promoter), GABA (*unc-47* promoter), pan-neuron (*snb-1* promoter), intestine (*vha-6* promoter).

Changes in GABA secretion could also contribute to the aldicarb resistance observed in *ric-7* mutants [22], [27], [28]. Consistent with this idea, *ric-7* mutants had defects in defecation behavior that are similar to those observed in mutants with decreased GABA transmission (Figure 8A). Contraction of the intestinal muscles during defecation (i.e. the expulsion step of the defecation motor program) is mediated by excitatory GABAergic input from defecation motor neurons [34]. We found that *ric-7* mutants had a significant expulsion defect, which was rescued by *ric-7* transgenes expressed in GABA neurons (Figure 8A). These results suggest that *ric-7* mutants have a presynaptic defect at GABAergic NMJs involved in defecation.

To assay GABA release at ventral cord NMJs (which are involved in locomotion), we recorded inhibitory post-synaptic currents (IPSCs) from body muscles (Figure 8B). We found that *ric-7* mutants had a wild type IPSC rate, while IPSC amplitudes were significantly increased (∼30%, p<0.001). These results suggest that GABA secretion still occurs in *ric-7* mutants, albeit in a subtly altered form. Changes in IPSC amplitudes are often caused by changes in post-synaptic sensitivity, e.g. by changing GABA receptor abundance. However, neither the current evoked by applying an exogenous GABA agonist nor the abundance of GFP-tagged UNC-49 GABA-A receptors were altered in *ric-7* mutants, suggesting that a post-synaptic defect was unlikely to account for the altered IPSC amplitude (Figure 3B, 3D). Consistent with a pre-synaptic defect, the distribution of GFP-tagged SNB-1 was altered in GABAergic neurons of *ric-7* mutants. Although SNB-1 puncta intensity was unaltered, puncta were significantly wider (23% wider, *p*<0.001) and diffuse SNB-1 axon fluorescence was significantly increased in *ric-7* mutants (Figure 8C). Finally, the IPSC and SNB-1 defects were both rescued by transgenes expressing RIC-7 in GABAergic motor neurons (Figure 8B–8C). Collectively, these results support the idea that *ric-7* mutants have a pre-synaptic defect that increases GABA secretion, perhaps by increasing the amount of GABA packaged into each SV. Rescue of the IPSC defect (with *ric-7* transgenes expressed in GABA neurons) failed to rescue the *ric-7* aldicarb defect and weakly rescued the locomotion defect implying that the latter were not caused by altered GABA secretion (Figure 1C).

Mutations disrupting neuropeptide signaling exhibit defecation defects similar to *ric-7* mutants [25], [35]. Prompted by these results, we wondered if the Ric-7 defecation and IPSC defects are caused by the neuropeptide secretion defect. In this scenario, we would expect that *ric-7* mutations and mutations that impair proneuropeptide processing would not have additive effects on defecation behavior in double mutants. Contrary to this idea, the defecation defects observed in *ric-7; egl-21* double mutants were significantly worse than those observed in either single mutant (Figure 8A). In addition, IPSC amplitudes were unaltered in *egl-3; egl-21* doubles mutants (Figure S1), suggesting that decreased neuropeptide secretion is also unlikely to explain the Ric-7 IPSC defect. These results indicate that RIC-7 cell-autonomously regulates two forms of secretion, promoting neuropeptide secretion and inhibiting GABA secretion.

## Discussion

Communication between neurons and their targets relies on two classes of neurosecretory vesicles, synaptic vesicles (SVs) and dense-core vesicles (DCVs). The mechanisms governing SV and DCV secretion share many properties (including related SNARE proteins, SNARE binding proteins, and calcium sensors). Despite these similarities, SV and DCV mediated secretion also have significant differences [1], which imply that some proteins will be selectively utilized in one or the other process. Because neuropeptides have dramatic effects on personality, behavior, and metabolism, there is significant interest in identifying molecules that selectively promote DCV secretion. Here we identify RIC-7 as a novel protein that is required for DCV secretion but has relatively subtle effects on SV secretion. Below we discuss the implications of these findings.

The *ric-7* gene was identified in a screen for mutations conferring resistance to aldicarb induced paralysis. As RIC-7 lacks identified structural or functional motifs, the mechanisms underlying this behavioral defect were unclear. In principle, aldicarb resistance could arise from several alternative mechanisms, including: altered muscle responsiveness to ACh or GABA, decreased excitatory input from cholinergic motor neurons, increased hyperpolarizing input from GABA motor neurons, or decreased neuropeptide signaling. Our results strongly support the idea that Ric-7 aldicarb and locomotion defects arise from disruption of neuropeptide secretion.

Several results suggest that RIC-7 does not regulate muscle sensitivity to neuromuscular agonists. The currents evoked by applying exogenous ACh and GABA to body muscles, and the expression of nicotinic and GABA receptors in body muscles were both unaffected in *ric-7* mutants. Furthermore, *ric-7* aldicarb and locomotion defects were not corrected by transgenes expressing RIC-7 in body muscles, whereas rescue was observed for transgenes expressed in cholinergic neurons. Thus, RIC-7 neither acts in body muscles, nor regulates muscle sensitivity to agonists.

Other results indicate that RIC-7 is not required for baseline ACh secretion. In cholinergic motor neurons, the SV protein SNB-1 neither accumulated in synaptic puncta nor in the axonal membrane (as would occur in exocytosis and endocytosis mutants, respectively). The rate, amplitude, and kinetics of endogenous and evoked EPSCs were unaltered in *ric-7* mutants. Thus, although RIC-7 expression in cholinergic neurons rescued the aldicarb and locomotion defects of *ric-7* mutants, these defects are unlikely to arise from decreased baseline ACh secretion.

In GABA neurons, *ric-7* mutants had a presynaptic defect that led to an apparent increase in GABA secretion. This presynaptic defect was manifest by an increase in amplitude of endogenous IPSCs in *ric-7* mutants, while the IPSC rate was unaltered. Changes in IPSC amplitudes are often caused by changes in post-synaptic sensitivity, e.g. by changing GABA receptor abundance. However, neither the current evoked by applying an exogenous GABA agonist nor the abundance of GFP-tagged UNC-49 GABA-A receptors were altered in *ric-7* mutants, suggesting that a post-synaptic defect was unlikely to account for the altered IPSC amplitude. Consistent with a pre-synaptic defect, SNB-1 axonal fluorescence was significantly increased in *ric-7* mutant GABA motor neurons. The defecation, IPSC, and SNB-1 defects were all rescued by transgenes expressing RIC-7 in GABAergic motor neurons. Collectively, these results support the idea that *ric-7* mutants have a pre-synaptic defect that increases GABA secretion. Rescue of the IPSC defect did not correlate with rescue of *ric-7* aldicarb and locomotion defects, implying that the latter were not caused by altered GABA secretion. In principle, the IPSC defect could cause the Ric-7 defecation defect, for example if increased GABA secretion causes constitutive excitation of the intestinal muscles. Double mutant analysis suggests that decreased neuropeptide secretion is unlikely to account for the Ric-7 IPSC and defecation defects. Consequently, our results are most consistent with the idea that RIC-7 has direct cell autonomous effects on two forms of secretion, promoting neuropeptide secretion and inhibiting GABA secretion. Several other proteins are known to have distinct effects on different neurotransmitter systems. Mouse Munc13-1 knockouts drastically reduce glutamatergic transmission yet have little effect on GABA release [36]. Mouse ELKS2 knockouts have increased GABA release but unaltered glutamate release [37]. Finally, complexin promotes evoked release but inhibits tonic/spontaneous release [38], [39].

Several results suggest that the effects of RIC-7 on aldicarb sensitivity were caused by changes in neuropeptide secretion. First, mutations preventing neuropeptide processing (*egl-21* CPE, *egl-3* PC2) and *ric-7* mutations did not have additive effects on aldicarb responsiveness in double mutants. Second, *ric-7* mutants had greatly decreased secretion of YFP-tagged neuropeptides (NLP-21 and INS-22) from cholinergic motor neurons. Third, mCherry-tagged RIC-7 co-localized extensively with a DCV marker (NLP-21), implying that RIC-7 could play a relatively direct role in regulating neuropeptide release. Fourth, *ric-7* mutants have decreased aldicarb-evoked secretion of NLP-12 from DVA neurons and lack aldicarb-induced potentiation of evoked ACh release (which is mediated by endogenous NLP-12). And fifth, restoring RIC-7 expression in DVA neurons was sufficient to partially rescue the Ric-7 aldicarb-resistance defect. Taken together, these results strongly support the idea that RIC-7 promotes secretion of endogenous neuropeptides, and that this function plays an important role in the Ric-7 aldicarb resistance and locomotion defects. The Ric-7 aldicarb resistance and locomotion defects are more severe than those observed in mutants lacking NLP-12 or those lacking EGL-3 PC2, indicating that additional RIC-7 functions also contribute to these phenotypes. These additional functions could include promoting secretion of other neuropeptides or novel RIC-7 functions that are not yet defined.

What aspect of DCV secretion is regulated by RIC-7? Decreased neuropeptide secretion in *ric-7* mutants could reflect changes in any aspect of DCV biogenesis, transport, docking, priming, calcium-triggering, or fusion. Several results suggest that RIC-7 is not packaged into DCVs, and consequently cannot play a role in pro-neuropeptide processing. RIC-7 lacks a predicted signal peptide sequence, GFP-tagged RIC-7 is not secreted, and RIC-7 delivery to axons is not prevented in *unc-104* KIF1A mutants. Collectively, these results suggest RIC-7 is cytoplasmic, and thus cannot play a direct role in neuropeptide processing. DCV biogenesis and transport also occur normally in *ric-7* mutants, as neuropeptide fluorescence in motor axons was increased rather than decreased. These results suggest that RIC-7 regulates a step that occurs after DCV transport. Given the prominent colocalization of RIC-7 and DCV markers in axons, we speculate the RIC-7 could identify DCV release sites. Because the sequence of RIC-7 does not provide any clues as to its biochemical function, further experiments will be required to determine a more precise function for RIC-7. Understanding the mechanisms underlying RIC-7's functions will undoubtedly shed light on how DCVs assume their unique properties.

The mechanisms governing SV and DCV secretion share many properties, including: SNARE proteins, SNARE binding proteins (e.g. Munc13, Munc18, and CAPS), and calcium sensors (e.g. Synaptotagmin) [40]. These shared mechanisms are highly conserved across eukaryotic phylogeny, suggesting that these core exocytosis components comprise an ancient process. By contrast, RIC-7 orthologs are observed in other nematodes but homologous genes are not detected in other sequenced genomes. Interestingly, other putative DCV secretion factors have similar patterns of conservation. For example, the Rab27 effector granuphilin is conserved in mammals and flies but not in *C. elegans*, while a second Rab27 effector melanophilin is present in mammals but absent in flies and worms. These results suggest that the mechanisms distinguishing SV and DCV secretion evolved more recently than the more ancient shared secretion factors.

## Materials and Methods

### *C. elegans* strains and drug assays

Strains were maintained at 20°C as described [41]. The wild-type reference strain was N2 Bristol. Descriptions of allele lesions can be found at http://www.wormbase.org. The mutant strains used were: LGIV, *egl-21(n476)*; LGV, *egl-3(nr2090), ric-7 (nu447), ric-7(n2657)*. Acute aldicarb and levamisole assays were performed blind in triplicate on young adult worms as described [42]. The aldicarb (Chem Services) concentrations used ranged from 1 to 2 mM, the levamisole (Sigma) concentration was 200 µM. Locomotion was measured by transferring adults to plates containing fresh (16 h) lawns of HB101 bacteria, letting the worms recover for 30 min and counting the number of body bends of active worms in a 3 min interval.

### *ric-7* mapping and cloning

*ric-7(nu447)* was isolated in an EMS screen for suppressors of the aldicarb hypersensitive phenotype of *dgk-1(nu62)* mutants (D.S. and J.K., unpublished data). *nu447* was mapped by small nucleotide polymorphism (SNP) mapping, based on its aldicarb resistance phenotype. Analysis of 16 *nu447*/CB4856 recombinants mapped *nu447* to LGV, map unit 5∼6; Another 62 clones further positioned *nu447* to 5.86∼6.00 m.u. Seven predicted genes were found in this interval with three of them previously characterized. Sequencing revealed that *ric-7(nu447)* contained a 830C/T (A277V) point mutation plus a 32 bp deletion (nucleotides 910–941) in F58E10.1b cDNA that shifts the reading frame, leading to a truncated RIC-7 protein that contains 305 amino acids. *ric-7(n2657)* contained a 621G/A (W207Stop) nonsense mutation in F58E10.1b cDNA. *nu447* was backcrossed six times and used for most phenotypic analysis in this study.

### Fluorescence microscopy and quantitative analysis

All quantitative imaging was done using a Olympus PlanAPO 100×1.4 NA objective and an ORCA100 CCD camera (Hamamatsu). Worms were immobilized with 30 mg/ml BDM (Sigma). Image stacks were captured and maximum intensity projections were obtained using Metamorph 7.1 software (Molecular Devices). GFP fluorescence was normalized to the absolute mean fluorescence of 0.5 mm FluoSphere beads (Molecular Probes). For dorsal cord imaging, young adult worms, in which the dorsal cords were oriented toward the objective, were imaged in the region midway between the posterior gonad bend at the tail. Line scans of dorsal cord fluorescence were analyzed in Igor Pro (WaveMetrics) using custom-written software [43], [44]. For coelomocyte imaging, the posterior coelomocyte was imaged in young adults [20]. Image stacks were captured and maximum intensity projections were obtained using Metamorph 7.1 software (Molecular Devices). For quantitation, the five brightest vesicles were analyzed for each coelomocyte and the mean fluorescence for each vesicle was logged. For each worm, coelomocyte fluorescence was calculated as the mean of the vesicle values in that animal. All p-values indicated were based on student t-tests.

Confocal images were taken using the Olympus FV1000 confocal microscope. Image stacks were captured, and maximum intensity projections were obtained using Metamorph 7.1 software (Universal Imaging).

### Electrophysiology

Electrophysiology was done on dissected adults as previously described [45]. All recording conditions were as described previously [20]. For comparing average electrophysiological values, statistical significance was determined using the Mann-Whitney test or student's t test.

### RIC-7 constructs and transgenes

Transgenic strains were generated by injecting either wild type or *ric-7(nu447)* mutants with the expression construct (10–25 ng/µl) mixed with the co-injection markers, KP#1338 (*pttx-3::GFP*), KP#1480 (*pmyo-2::NLS-mCherry*) or KP#1106 (*pmyo-2::NLS-GFP*), each at 10 ng/µl, using standard methods [46]. The co-localization experiments were done by co-injecting KP#1684 (*pric-7::his-24 cDNA::wcherry*) with KP#1685 (*punc-30::NLS-GFP*) or with KP#1686 (*punc-17::NLS-GFP*), respectively; or by injecting KP#1687 (*punc-129::RIC-7 cDNA::GFP*) into *nuIs252 (Punc-129::mchry::PaGFP::unc-57)*, or into *nuIs470* (*punc-129::NLP-21::mCherry*), respectively, all at a concentration of 25 ng/µl each. All constructs except KP#1684 were derivatives of pPD49.26 [47].

Two RIC-7 constructs rescued the *ric-7(nu447)* mutant aldicarb defect: KP#1680 (*psnb-1::RIC-7*), and KP#1681 (*punc-17::RIC-7*). Two RIC-7 constructs did not rescue the *ric-7(nu447)* mutant aldicarb defect: KP #1682(*punc-25::RIC-7*), and KP#1683(*pmyo-3::RIC-7*). All four constructs used F58E10.1b cDNA cloned with NheI and KpnI sites.

The previously reported lines used for imaging experiments described are *nuIs152 (punc-129::GFP::SNB-1)*, *nuIs183 (punc-129::NLP-21::VENUS), nuIs195 (punc-129::INS-22::VENUS)* [20], [24], *nuIs283 (pmyo-3::UNC-49::GFP)* (J. Bai and J.K., unpublished), *nuIs299 (pmyo-3::ACR-16::GFP)* [48], *nuIs444 (pnlp-12::NLP-12::VENUS)* [31] and *nuIs376 (punc-25::SNB-1::GFP)*.

## Supporting Information

Figure S1 IPSCs are not altered in neuropeptide processing mutants. Endogenous IPSCs were recorded from adult body wall muscles of the indicated genotypes. Representative traces (A), and summary data (B) are shown. The number of animals analyzed is indicated for each genotype. Error bars indicate SEM. No significant differences were observed.
(TIF)

## References

[1] Burgoyne RD, Morgan A (2003). Secretory granule exocytosis.. Physiol Rev.

[2] Sudhof TC (2004). The synaptic vesicle cycle.. Annu Rev Neurosci.

[3] Weimer RM, Richmond JE, Davis WS, Hadwiger G, Nonet ML (2003). Defects in synaptic vesicle docking in unc-18 mutants.. Nat Neurosci.

[4] Voets T, Toonen RF, Brian EC, de Wit H, Moser T (2001). Munc18-1 promotes large dense-core vesicle docking.. Neuron.

[5] Hammarlund M, Palfreyman MT, Watanabe S, Olsen S, Jorgensen EM (2007). Open syntaxin docks synaptic vesicles.. PLoS Biol.

[6] Hammarlund M, Watanabe S, Schuske K, Jorgensen EM (2008). CAPS and syntaxin dock dense core vesicles to the plasma membrane in neurons.. J Cell Biol.

[7] de Wit H, Cornelisse LN, Toonen RF, Verhage M (2006). Docking of secretory vesicles is syntaxin dependent.. PLoS One.

[8] Klenchin VA, Martin TF (2000). Priming in exocytosis: attaining fusion-competence after vesicle docking.. Biochimie.

[9] Martin TF (2002). Prime movers of synaptic vesicle exocytosis.. Neuron.

[10] Jena BP (2005). Cell secretion and membrane fusion.. Domest Anim Endocrinol.

[11] Sorensen JB (2005). SNARE complexes prepare for membrane fusion.. Trends Neurosci.

[12] Voets T, Moser T, Lund PE, Chow RH, Geppert M (2001). Intracellular calcium dependence of large dense-core vesicle exocytosis in the absence of synaptotagmin I.. Proc Natl Acad Sci U S A.

[13] Edwards RH (1998). Neurotransmitter release: variations on a theme.. Curr Biol.

[14] Kim T, Gondre-Lewis MC, Arnaoutova I, Loh YP (2006). Dense-core secretory granule biogenesis.. Physiology (Bethesda).

[15] Bruns D, Jahn R (1995). Real-time measurement of transmitter release from single synaptic vesicles.. Nature.

[16] Grishanin RN, Kowalchyk JA, Klenchin VA, Ann K, Earles CA (2004). CAPS acts at a prefusion step in dense-core vesicle exocytosis as a PIP2 binding protein.. Neuron.

[17] Berwin B, Floor E, Martin TF (1998). CAPS (mammalian UNC-31) protein localizes to membranes involved in dense-core vesicle exocytosis.. Neuron.

[18] Speese S, Petrie M, Schuske K, Ailion M, Ann K (2007). UNC-31 (CAPS) is required for dense-core vesicle but not synaptic vesicle exocytosis in Caenorhabditis elegans.. J Neurosci.

[19] Jockusch WJ, Speidel D, Sigler A, Sorensen JB, Varoqueaux F (2007). CAPS-1 and CAPS-2 are essential synaptic vesicle priming proteins.. Cell.

[20] Sieburth D, Madison JM, Kaplan JM (2007). PKC-1 regulates secretion of neuropeptides.. Nat Neurosci.

[21] Kang L, He Z, Xu P, Fan J, Betz A (2006). Munc13-1 is required for the sustained release of insulin from pancreatic beta cells.. Cell Metab.

[22] Miller KG, Alfonso A, Nguyen M, Crowell JA, Johnson CD (1996). A genetic selection for Caenorhabditis elegans synaptic transmission mutants.. Proc Natl Acad Sci U S A.

[23] Nguyen M, Alfonso A, Johnson CD, Rand JB (1995). Caenorhabditis elegans mutants resistant to inhibitors of acetylcholinesterase.. Genetics.

[24] Sieburth D, Ch'ng Q, Dybbs M, Tavazoie M, Kennedy S (2005). Systematic analysis of genes required for synapse structure and function.. Nature.

[25] Jacob TC, Kaplan JM (2003). The EGL-21 carboxypeptidase E facilitates acetylcholine release at Caenorhabditis elegans neuromuscular junctions.. J Neurosci.

[26] Bai J, Hu Z, Dittman JS, Pym EC, Kaplan JM (2010). Endophilin functions as a membrane-bending molecule and is delivered to endocytic zones by exocytosis.. Cell.

[27] Vashlishan AB, Madison JM, Dybbs M, Bai J, Sieburth D (2008). An RNAi screen identifies genes that regulate GABA synapses.. Neuron.

[28] Mullen GP, Mathews EA, Saxena P, Fields SD, McManus JR (2006). The Caenorhabditis elegans snf-11 gene encodes a sodium-dependent GABA transporter required for clearance of synaptic GABA.. Mol Biol Cell.

[29] Dittman J, Kaplan J (2006). Factors regulating the abundance and localization of Synaptobrevin in the plasma membrane.. PNAS.

[30] Ch'ng Q, Sieburth D, Kaplan JM (2008). Profiling synaptic proteins identifies regulators of insulin secretion and lifespan.. PLoS Genet.

[31] Hu Z, Pym EC, Babu K, Vashlishan Murray AB, Kaplan JM (2011). A neuropeptide-mediated stretch response links muscle contraction to changes in neurotransmitter release.. Neuron.

[32] Janssen T, Meelkop E, Lindemans M, Verstraelen K, Husson SJ (2008). Discovery of a cholecystokinin-gastrin-like signaling system in nematodes.. Endocrinology.

[33] Li W, Feng Z, Sternberg PW, Xu XZ (2006). A C. elegans stretch receptor neuron revealed by a mechanosensitive TRP channel homologue.. Nature.

[34] Jorgensen EM (2005). Gaba.. WormBook.

[35] Mahoney TR, Luo S, Round EK, Brauner M, Gottschalk A (2008). Intestinal signaling to GABAergic neurons regulates a rhythmic behavior in Caenorhabditis elegans.. Proc Natl Acad Sci U S A.

[36] Augustin I, Rosenmund C, Sudhof TC, Brose (1999). Munc13-1 is essential for fusion competence of glutamatergic synaptic vesicles [In Process Citation].. Nature.

[37] Kaeser PS, Deng L, Chavez AE, Liu X, Castillo PE (2009). ELKS2alpha/CAST deletion selectively increases neurotransmitter release at inhibitory synapses.. Neuron.

[38] Martin JA, Hu Z, Fenz KM, Fernandez J, Dittman JS (2011). Complexin has opposite effects on two modes of synaptic vesicle fusion.. Curr Biol.

[39] Hobson RJ, Liu Q, Watanabe S, Jorgensen EM (2011). Complexin maintains vesicles in the primed state in C. elegans.. Curr Biol.

[40] Xu T, Xu P (2008). Searching for molecular players differentially involved in neurotransmitter and neuropeptide release.. Neurochem Res.

[41] Brenner S (1974). The genetics of Caenorhabditis elegans.. Genetics.

[42] Lackner MR, Nurrish SJ, Kaplan JM (1999). Facilitation of synaptic transmission by EGL-30 Gqalpha and EGL-8 PLCbeta: DAG binding to UNC-13 is required to stimulate acetylcholine release.. Neuron.

[43] Burbea M, Dreier L, Dittman JS, Grunwald ME, Kaplan JM (2002). Ubiquitin and AP180 regulate the abundance of GLR-1 glutamate receptors at postsynaptic elements in C. elegans.. Neuron.

[44] Dittman JS, Kaplan JM (2006). Factors regulating the abundance and localization of synaptobrevin in the plasma membrane.. Proc Natl Acad Sci U S A.

[45] Richmond JE, Jorgensen EM (1999). One GABA and two acetylcholine receptors function at the C. elegans neuromuscular junction.. Nat Neurosci.

[46] Mello CC, Kramer JM, Stinchcomb D, Ambros V (1991). Efficient gene transfer in C.elegans: extrachromosomal maintenance and integration of transforming sequences.. Embo J.

[47] Fire A (1997). Fire Vector Kit..

[48] Simon DJ, Madison JM, Conery AL, Thompson-Peer KL, Soskis M (2008). The microRNA miR-1 regulates a MEF-2-dependent retrograde signal at neuromuscular junctions.. Cell.

