# Supplementary figures and images for: RIC-7 Promotes Neuropeptide Secretion
Source: PLoS Genet. 2012 Jan 19;8(1):e1002464. doi: 10.1371/journal.pgen.1002464 (PMC3261915; doi:10.1371/journal.pgen.1002464)

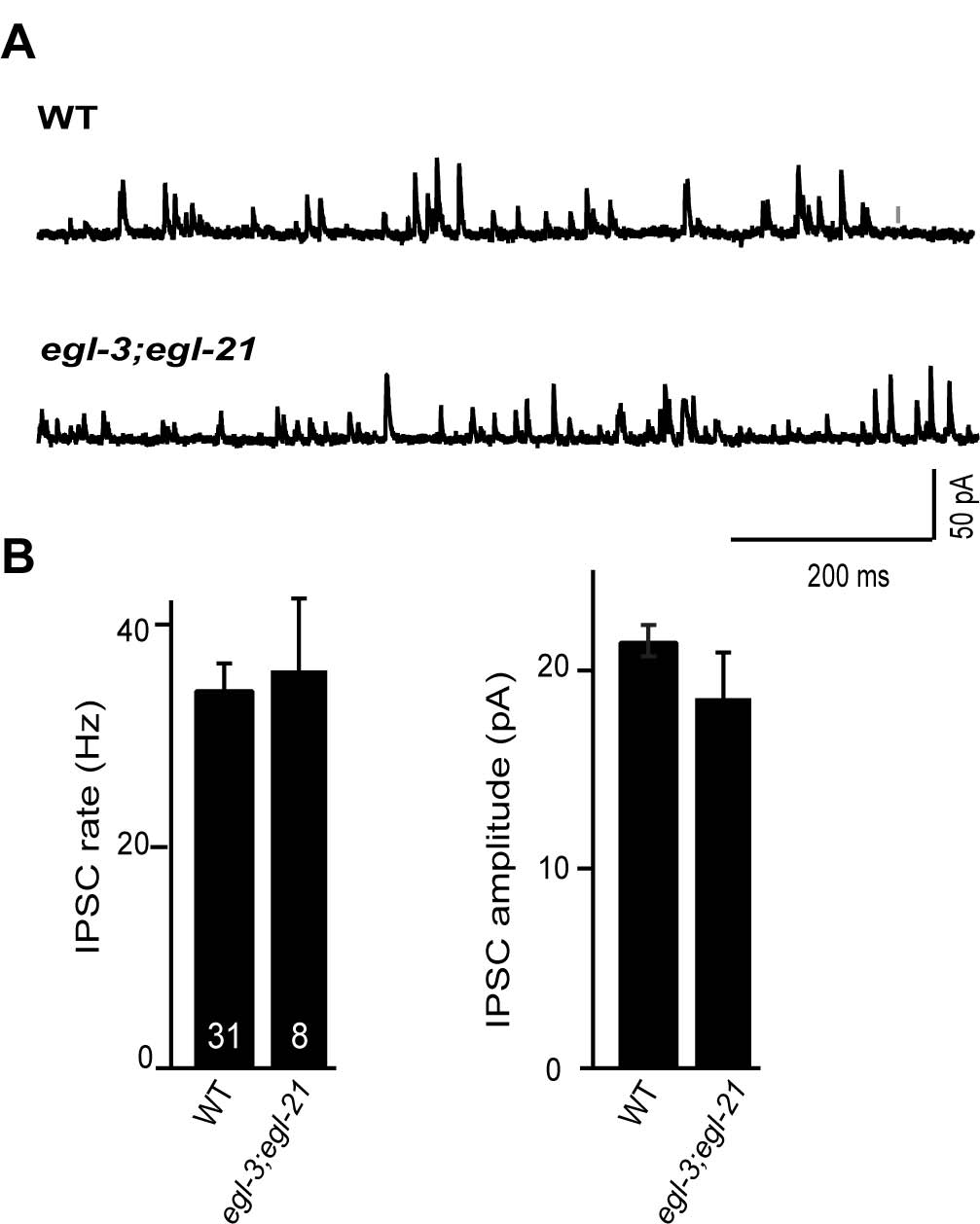

Supplement: Figure S1 — IPSCs are not altered in neuropeptide processing mutants. Endogenous IPSCs were recorded from adult body wall muscles of the indicated genotypes. Representative traces (A), and summary data (B) are shown. The number of animals analyzed is indicated for each genotype. Error bars indicate SEM. No significant differences were observed. (TIF) [file pgen.1002464.s001.tif]
